# Prognostic Nomograms for Hospital Survival and Transplant-Free Survival of Patients with Hepatorenal Syndrome: A Retrospective Cohort Study

**DOI:** 10.3390/diagnostics12061417

**Published:** 2022-06-08

**Authors:** Yi Song, Yu Wang, Chaoran Zang, Xiaoxi Yang, Zhenkun Li, Lina Wu, Kang Li

**Affiliations:** 1 Institute of Clinical Medicine, Beijing Friendship Hospital, Capital Medical University, Beijing 100050, China; songmiaoxi@126.com (Y.S.); yangxx999999999@163.com (X.Y.); iceblade7@163.com (Z.L.); 2 Liver Research Center, Beijing Friendship Hospital, Capital Medical University, Beijing 100050, China; wangyuliver@ccmu.edu.cn; 3 Hepatobiliary Pancreatic Center Department, Beijing Tsinghua Changgung Hospital Affiliated to Tsinghua University, Beijing 102218, China; zangchaoran2012@163.com; 4 Biomedical Information Center, Beijing You’ An Hospital, Capital Medical University, Beijing 100069, China

**Keywords:** hepatorenal syndrome, hospital survival, transplant-free survival, retrospective cohort, nomogram

## Abstract

Hepatorenal syndrome (HRS) is a life-threatening complication of cirrhosis with a poor prognosis. To develop novel and effective nomograms which could numerically predict both the hospital survival and transplant-free survival of HRS, we retrospectively enrolled a cohort of 149 patients. A backward stepwise method based on the smallest Akaike information criterion value was applied to select the covariates to be included in the Cox proportional hazards models. The Harrell C-index, area under the receiver operating characteristic curve (AUC), Brier score, and Kaplan–Meier curves with the log-rank test were used to assess nomograms. The bootstrapping method with 1000 resamples was performed for internal validation. The nomogram predicting hospital survival included prothrombin activity, HRS clinical pattern, Child–Pugh class, and baseline serum creatinine. The C-index was 0.72 (95% confidence interval (CI), 0.65–0.78), and the adjusted C-index was 0.72 (95% CI, 0.66–0.79). The nomogram predicting transplant-free survival included sex, prothrombin activity, HRS clinical pattern, model for end-stage liver disease–Na score, and peak serum creatinine. The C-index of the nomogram was 0.74 (95% CI, 0.69–0.79), and the adjusted C-index was 0.74 (95% CI, 0.68–0.79). The AUC and Brier score at 15, 30, and 45 days calculated from the hospital survival nomogram and those at 6, 12, and 18 months calculated from the transplant-free survival nomogram revealed good predictive ability. The two models can be used to identify patients at high risk of HRS and promote early intervention treatment.

## 1. Introduction

Hepatorenal syndrome (HRS) is a severe impairment of kidney function, and it occurs in patients with end-stage liver disease. HRS is typically classified into two clinical patterns. In the first pattern, there is an abrupt impairment of kidney function termed HRS-acute kidney injury (AKI), historically known as type 1 HRS. HRS-AKI is a type of AKI, and it is common in patients with decompensated cirrhosis who are absent of hypovolemia or structural kidney injury. The prevalence of AKI in hospitalized patients with decompensated cirrhosis ranges between 27% and 53% [1]. In previous studies, HRS-AKI was reported to account for 15–43% of AKI [1]. In the second pattern, there is chronic impairment of kidney function termed HRS-chronic kidney disease (CKD), historically known as type 2 HRS. Because of a reduction in the glomerular filtration rate (GFR), HRS-CKD belongs to the CKD category.

Several randomized controlled trials and meta-analyses have shown that vasoconstrictors combined with albumin are effective in improving kidney function in patients with HRS [2]. The response rate was found to range from 20% to 80% [1]. Renal replacement therapy (RRT) is considered when kidney function progressively deteriorates, severe acidosis occurs, hyperkalemia does not improve with medical management, there is an increase in volume overload, or as a bridge to liver transplantation (LT) for transplant candidates. LT is the ultimate therapy for HRS-AKI. However, the mortality rate is still significantly high in HRS-AKI, even after evidence-based medical treatment, and many factors may impact the recovery of the kidney after LT [3,4].

HRS is associated with a poor prognosis in patients with cirrhosis. The median survival times of patients with traditional type 2 HRS and type 1 HRS without pharmacological treatment or LT were found to be six months and one month, respectively [5]. Patients with severe or repeated episodes of AKI have been found to be at high risk of developing CKD [6]. The 30-day mortality rate of patients with HRS-AKI ranges from 29% to 44% [7]. AKI has also been found to be an independent negative predictor of hospital survival [8,9,10], mid-term survival [11], transplant-free survival, and post-LT outcomes in patients with decompensated cirrhosis. Therefore, it is critical to effectively and accurately evaluate and predict mortality.

The Child–Pugh score has been widely used to evaluate the hepatic reserve capacity. The model for end-stage liver disease (MELD)/MELD–Na score includes serum creatinine (sCr), which serves as one of the main determinants of the score, and priority is given to LT when it is applied to a prognostic prediction in cases of decompensated cirrhosis with HRS [12]. In addition, some studies have focused on the prognostic prediction of HRS. Age, serum bilirubin, and response after fluid replacement therapy were found to be independent predictors of mortality in HRS [13]. In another study, baseline bilirubin, the lack of reversibility of HRS, unresolved infection, and sepsis were associated with a poor prognosis of HRS-AKI [14]. A previous study provided a prognostic model score system to predict the 28-day mortality of patients with HRS [15]. Previous reports have only focused on either short-term survival or long-term survival. Thus, new comprehensive prognostic models must urgently be developed to predict the hospital survival and transplant-free survival of patients with HRS. In this study, we developed novel, effective and convenient nomograms which could numerically predict both the hospital survival and transplant-free survival for patients with HRS.

## 2. Materials and Methods

### 2.1. Cohort Construction

This was a single-center, retrospective cohort study enrolling 343 patients with decompensated cirrhosis from the Beijing Friendship Hospital from February 2013 to December 2021. The demographic data, medical history, physical examination data, relevant laboratory data (including complete blood count, liver function tests, renal function tests, serum electrolytes, coagulation profile, and ascetic fluid analysis) of the patients were obtained from the hospital information system (HIS). Doppler abdominal ultrasonography findings were collected to rule out hepatocellular carcinoma (HCC) and to evaluate the grading of ascites. A value of sCr obtained in the previous three months, or that obtained closest to or at admission to hospital, was defined as the baseline sCr level. We calculated the Child–Pugh score and MELD–Na score based on laboratory test results within 24 h of hospital admission.

The studies involving human participants were reviewed and approved by the Ethics Committee of the Beijing Friendship Hospital, Approval No. 2022-P2-006-01. Given its retrospective nature and that only anonymous data was analyzed, no additional patient informed consent that was specific to this study was required. This study followed the recommendations described by the Declaration of Helsinki.

Inclusion and exclusion criteria: The inclusion criteria were as follows: (1) Decompensated cirrhosis with ascites. (2) HRS-AKI defined as an increase in sCr ≥ 26.5 μmol/L within 48 h or a percentage increase in sCr ≥ 50% within the prior seven days, according to the International Club of Ascites. HRS-CKD was defined as a reduction in the estimated GFR < 60 mL/1.73 m^2^ per minute for at least 3 months [16,17]. (3) No response after two consecutive days of diuretic withdrawal and plasma volume expansion with albumin infusion. The exclusion criteria were as follows: (1) hypovolemia, (2) septic shock, (3) current or recent use of nephrotoxic drugs/agents, (4) HCC or other malignant tumors, and (5) aged under 18 years old.

Outcomes: The patients were followed-up to evaluate survival after HRS by contacting the patients or their family members, or by collecting information from the HIS. The outcomes of interest were hospital survival and transplant-free survival. Hospital survival was defined as survival without liver-related death at the time of discharged. Three patients were hospitalized for more than 60 days before undergoing LT and the three patients were included in the hospital survival analysis (*n* = 139). Transplant-free survival was defined as survival without liver-related death or LT during the 18-month follow-up. We included all patients (*n* = 149) in the transplant-free survival analysis and took into consideration of composite end point of liver-related death or LT.

### 2.2. Statistical Analysis

Continuous variables are reported as means and standard deviations (SDs) (normally distributed data) or medians with minimum number and maximum number (non-normally distributed data), and categorical variables are reported as whole numbers and proportions. A backward stepwise method based on the smallest Akaike information criterion (AIC) value was applied to select the covariates to be included in the Cox proportional hazards models. The hazard ratio (HR) was presented with its 95% confidence interval (CI). Nomograms were developed with coefficients of the selected variables. The Schoenfeld residuals test was applied to evaluate the proportional hazards assumption. The nomogram performance was assessed by discriminating ability and calibration. Discrimination was indicated by Harrell’s C-index and the area under the ROC curve (AUC), and calibration was measured using the Brier score. Bias-corrected calibration using the bootstrapping method with 1000 resamples was performed for internal validation of the nomograms. The survival probabilities of the patients in the study were evaluated using the Kaplan–Meier survival analysis, and the differences in survival probabilities were examined using the log-rank test.

R software (version 3.6.3) was used for all of the statistical analyses. R packages, including ggplot2, survival, rms, survminer, pec, plotROC, and riskRegression, were used to develop and validate the nomograms. The reported significance levels were all two-sided, with statistical significance set at 0.05.

## 3. Results

### 3.1. Demographic and Clinicopathological Characteristics

**Figure 1:**
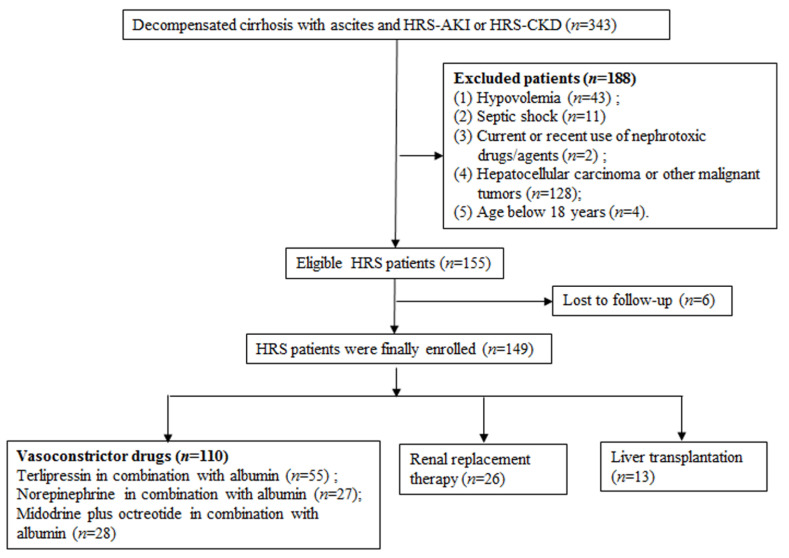
Flowchart of patient enrollment. Abbreviations: HRS, hepatorenal syndrome; AKI, acute kidney injury; CKD, chronic kidney disease.

We retrospectively enrolled 343 patients from the Beijing Friendship Hospital from February 2013 to December 2021. After the inclusion and exclusion criteria were applied, there were 155 eligible patients (Figure 1). Six patients were lost to follow-up. Finally, 149 patients with HRS remained for analysis, including 109 (73.15%) males, and the mean age was 55.01 ± 12.71 years. Most of the patients were determined to have cirrhosis caused by viral hepatitis (65, 43.62%). HRS-AKI (128, 85.90%) was the main clinical pattern, and 129 (86.58%) patients were rated as C class with the Child–Pugh score. Some patients had spontaneous bacterial peritonitis (17, 11.40%) or gastrointestinal bleeding (43, 28.86%). The median baseline sCr was 90.5 μmol/L (34.5–354.2), and the median peak sCr was 211.5 μmol/L (136.2–680.6). The use of vasoconstrictor drugs was the main treatment strategy, including terlipressin in combination with albumin (55, 36.91%), norepinephrine in combination with albumin (27, 18.12%), and midodrine plus octreotide in combination with albumin (28, 18.79%). A total of 26 (17.45%) patients underwent renal replacement therapy, and only 13 (8.72%) underwent LT (Table 1).

**Figure 2:**
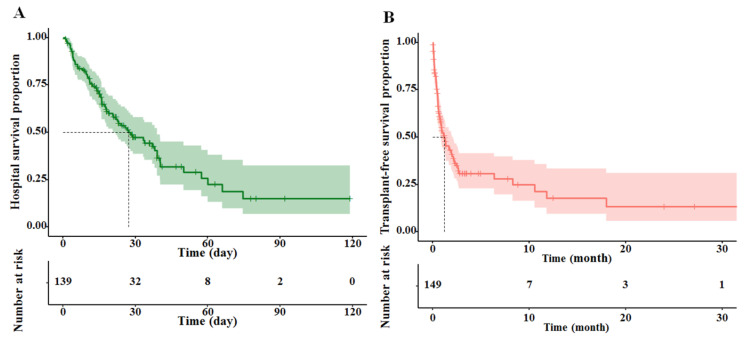
Kaplan–Meier curves demonstrating hospital survival (**A**) and transplant-free survival (**B**) with 95% confidence interval.

According to the Kaplan–Meier curves, the 15-, 30-, and 45-day hospital survival percentages were 70.20% (95% CI, 62.75–78.50%), 47.40% (95% CI, 38.71–58.10%), and 31.90% (95% CI, 22.50–45.20%), respectively. The 6-, 12-, and 18-month transplant-free survival percentages were 30.90% (95% CI, 22.90–41.5%), 17.80% (95% CI, 9.49–33.4%), and 13.40% (95% CI, 5.73–31.1%), respectively (Figure 2).

### 3.2. Model Specifications, and Predictors of Hospital Survival and Transplant-Free Survival

Univariate analyses showed that albumin, total bilirubin, prothrombin activity (PTA), the clinical pattern of HRS, Child–Pugh class, MELD–Na score, and baseline sCr were significantly correlated with hospital survival. A backward stepwise selection based on the AIC in the Cox proportional hazards regression multivariate analysis identified four independent prognostic factors: PTA, clinical pattern of HRS, Child–Pugh class, and baseline sCr (Table 2). Similarly, univariate analyses showed that sex, total bilirubin, PTA, clinical pattern of HRS, serum NH3, gastrointestinal bleeding, MELD–Na score, baseline sCr, and peak sCr were significantly correlated with transplant-free survival. A backward stepwise selection based on the AIC in the Cox proportional hazards regression multivariate analysis identified five independent prognostic factors: sex, PTA, HRS-AKI, MELD–Na score, and peak sCr (Table 2).

### 3.3. Development and Internal Validation of the Nomograms

**Figure 3:**
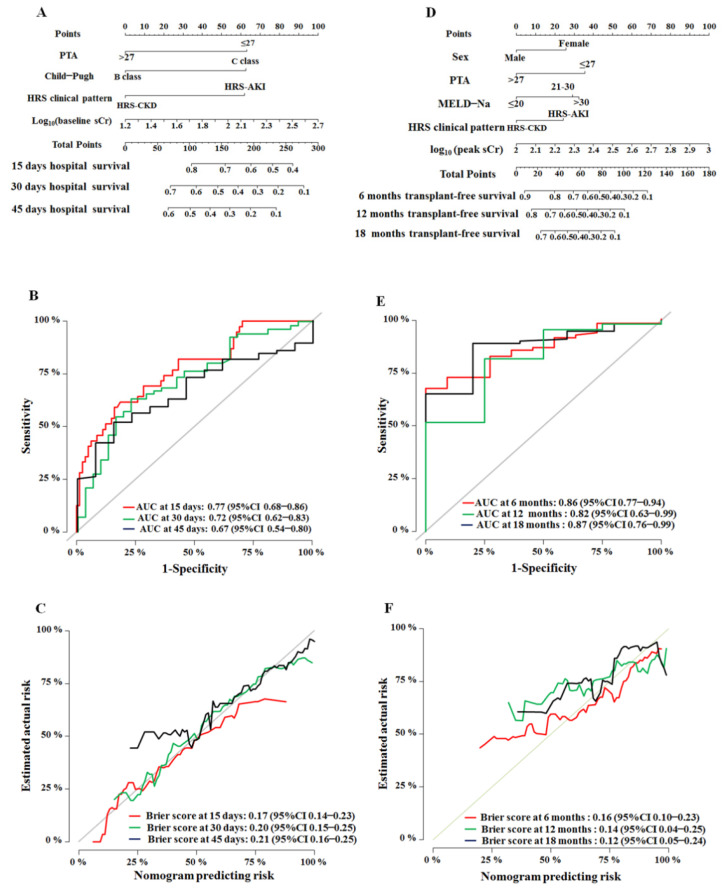
Development and internal validation of the hospital survival and transplant-free survival nomograms. (**A**) The hospital survival nomogram. The nomogram was based on four prognostic factors and could be used to predict the probability of hospital survival at 15, 30, and 45 days by adding up the points identified on the scales of these four parameters. (**B**) The time-dependent ROC curves and AUC at 15, 30, and 45 days are shown. (**C**) The Brier score calibration curves for the hospital survival nomogram at 15, 30, and 45 days. (**D**) The transplant-free survival nomogram. The nomogram was based on five prognostic factors and could be used to predict the probability of transplant-free survival at 6, 12, and 18 months. (**E**) The time-dependent ROC curves and AUC at 6, 12, and 18 months are shown. (**F**) The Brier score calibration curves for the transplant-free survival nomogram at 6, 12, and 18 months. Internal validation of the two nomograms was performed using the bootstrap sampling method. The time-dependent ROC curves were measured by bootstrapping with 1000 repetitions. A calibration curve developed using the bootstrap method with 1000 repetitions was used to estimate the probability at different times. The X-axis represents the predicted probability calculated from the nomogram, and the Y-axis represents the actual probability. Abbreviations: PTA: prothrombin activity; CKD: chronic kidney disease; AKI: acute kidney injury; sCr: serum creatinine; MELD–Na: model for end-stage liver disease–sodium; CI: confidence interval; AUC: area under the receiver operating characteristic curve; ROC: receiver operating characteristic.

The nomogram predicting hospital survival was created based on the following four independent prognostic factors: PTA (≤27 or >27), clinical pattern of HRS (HRS-AKI or HRS-CKD), Child–Pugh class (B or C), and baseline sCr (Figure 3A). All the individual and global Schoenfeld test *p* values were >0.05, indicating that each factor met the requirements for the proportional hazard (PH) test (Figure S1A). The discriminative ability of the model tested with the C-index was 0.72 (95% CI, 0.65–0.78). The nomogram predicting transplant-free survival was created based on the following five independent prognostic factors: sex (female or male), PTA (≤27 or >27), clinical pattern of HRS (HRS-AKI or HRS-CKD), MELD-Na score (≤20, 21–30 or >30), and peak sCr (Figure 3D). Moreover, all the individual and global Schoenfeld test *p* values were >0.05, and the factors met the requirements for the PH test (Figure S1B). The C-index of the nomogram was 0.74 (95% CI, 0.69–0.79).

The overfitting of the model was assessed using the bootstrap internal validation method. The adjusted C-indexes of the nomograms predicting hospital survival and transplant-free survival were 0.72 (95% CI, 0.66–0.79) and 0.74 (95% CI, 0.68–0.79), respectively, after 1000 bootstrap cross-validation iterations. AUC was used to validate the discriminative ability of the nomograms. The AUC values for the hospital survival nomogram at 15, 30, and 45 days were 0.77 (95% CI, 0.68–0.86), 0.72 (95% CI, 0.62–0.83), and 0.67 (95% CI, 0.54–0.80), respectively (Figure 3B). The adjusted Brier scores of the calibration curve for the nomogram at 15, 30, and 45 days were 0.17 (95% CI, 0.14–0.23), 0.20 (95% CI, 0.15–0.25), and 0.21 (95% CI, 0.16–0.25), respectively (Figure 3C). Similarly, the AUC values for the transplant-free survival nomogram at 6, 12, and 18 months were 0.86 (95% CI, 0.77–0.94), 0.82 (95% CI, 0.63–0.99), and 0.87 (95%CI, 0.76–0.99), respectively (Figure 3E), and the adjusted Brier scores were 0.16 (95% CI, 0.10–0.23), 0.14 (95% CI, 0.04–0.25), and 0.12 (95% CI, 0.05–0.24), respectively (Figure 3F).

### 3.4. Performance of Nomograms

**Figure 4:**
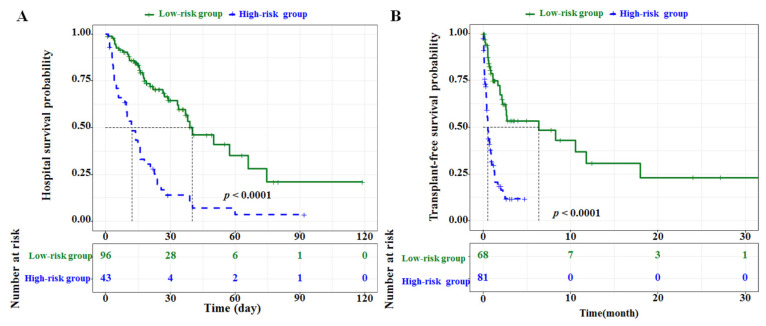
Kaplan–Meier curves of hospital survival (**A**) and transplant-free survival (**B**) according to low-risk or high-risk groups stratified by nomogram predictions. The *p* values were calculated using the log-rank test.

To further evaluate the discriminative ability of the two nomograms, the actual probabilities of hospital survival and transplant-free survival were plotted as Kaplan–Meier curves stratified by the high-risk and low-risk groups of the predicted probabilities calculated from the two nomograms (Figure 4A,B). According to the actual 15-, 30-, and 45-day hospital survival probabilities based on the Kaplan–Meier analysis, patients in the high-risk group had a lower hospital survival rate (40.70% (95% CI, 28.03–59.20%), 14.00% (95% CI, 6.32–31.00%), and 7.00% (95% CI, 1.98–24.7%), respectively) compared with patients in the low-risk group (83.40% (95% CI, 76.06–91.50%), 64.60% (95% CI, 54.23–76.90%), and 46.30% (95% CI, 33.37–64.20%), respectively (*p* < 0.001)) (Table 3). The 15-, 30-, and 45-day hospital survival probabilities predicted by the nomogram revealed good estimations of 41.25% (95% CI, 33.57–48.89%), 14.63% (95% CI, 9.41–19.84%), and 5.74% (95% CI, 2.74–8.74%) in the high-risk group, and 82.71% (95% CI, 80.93–85.33%), 63.70% (95% CI, 59.06–68.34%), and 49.36% (95% CI, 44.24–52.23%) in the low-risk group, respectively (*p* < 0.001) (Table 3). Similarly, based on the Kaplan–Meier analysis, the actual 6-, 12-, and 18-month transplant-free survival probabilities of the patients in the low-risk group were 53.4% (95% CI, 40.33–70.70%), 30.80% (95% CI, 16.55–57.40%), and 23.10% (95% CI, 9.97–53.60%), respectively. The 6-, 12-, and 18-month transplant-free survival probabilities predicted by the nomogram revealed good estimations of 56.50% (95% CI, 48.67–64.32%), 31.69% (95% CI, 22.93–40.52%), and 27.59% (95% CI, 19.03–36.16%) in the low-risk group, respectively (*p* < 0.001) (Table 3).

## 4. Discussion

HRS is a life-threatening complication in advanced liver cirrhosis and has a poor prognosis, even when evidence-based medical treatment is extensively used [18]. In this study, we developed and internally validated two nomograms that numerically predicted the hospital survival and transplant-free survival of patients with HRS. The prognostic information based on the two nomograms could be used to make decisions regarding early intervention treatment and surveillance. Previous reports have only focused on either short-term survival [15] or long-term survival [19]; however, we assessed both hospital survival and transplant-free survival in our cohort.

The nomogram used to predict hospital survival considered four prognostic factors, namely, PTA (≤27 or >27), the clinical pattern of HRS (HRS-AKI or HRS-CKD), Child–Pugh class (B or C), and peak sCr. Meanwhile, the nomogram used to predict transplant-free survival included sex (female or male), PTA (≤27 or >27), the clinical pattern of HRS (HRS-AKI or HRS-CKD), MELD–Na score (≤20, 21–30 or >30), and peak sCr. Our results demonstrate that extrinsic coagulation activity and the clinical pattern of HRS also played important prognostic roles in short-term and long-term survival [15,18,20,21,22]. Comparing patients with HRS-CKD, HRS-AKI had a poorer prognosis, and its main outcomes were hepatorenal failure and death. HRS-AKI had a higher short-term mortality, with a median survival of only two weeks [18]. Our results show that HRS-AKI also reduced the probability of long-term survival, and it was one of the prognostic factors (HR = 0.51, *p* = 0.045). We also considered the prognostic value of the peak sCr level in transplant-free survival. In a recent report, the peak sCr level was a risky prognostic predictor in patients with high-stage HRS-AKI after LT [23]. Similarly, the peak sCr level was a risky prognostic predictor in the transplant-free survival model in this study (HR = 14.72; *p* < 0.001).

The Child–Pugh score has been widely used to evaluate the hepatic reserve capacity, and patients with Child–Pugh C class have severe liver function impairment. It has been found that patients with traditional type 1 HRS and an elevated Child–Pugh score (>13) do not respond well to terlipressin [24]. In this study, a baseline sCr level was included in the hospital survival nomogram, which modified the limitation of the Child–Pugh score not accounting for renal function [25]. The MELD–Na score was initially used to predict 3- and 6-month mortality among patients with cirrhosis awaiting LT [26,27,28]. Moreover, it has now been widely validated as a prognosis predictor of survival in patients with liver diseases [29]. In this study, the MELD–Na score was included in the transplant-free survival nomogram, and a score of more than 20 was associated with high mortality (MELD–Na score = 21–30 (HR = 1.78; *p* = 0.078) and MELD–Na score > 30 (HR = 2.27; *p* = 0.005)). The sCr level played a pivotal prognostic role in renal function in HRS, and it was included in the MELD–Na score. An acute increase in the sCr level indicates the severity of renal dysfunction [30].

Our proposed nomograms demonstrated good discriminative ability, with a C-index of 0.72 (95% CI, 0.65–0.78) for the prediction of hospital survival and a C-index of 0.74 (95% CI, 0.69–0.79) for the prediction of transplant-free survival. In addition, the 15-, 30-, and 45-day hospital survival probabilities that were predicted by the hospital survival nomograms for the low-risk and high-risk groups were similar to those calculated with the Kaplan–Meier curves for the low-risk and high-risk groups. Similarly, the 6-, 12-, and 18-month transplant-free survival probabilities predicted by the transplant-free survival nomogram were similar to those calculated with the Kaplan–Meier curves for the low-risk group (Table 3).

In fact, HRS is rare, and it is a complication of advanced liver cirrhosis. In this study, we retrospectively enrolled 149 eligible patients to explore the prognostic information. An internal validation was performed using the resampling bootstrap method with 1000 repetitions, which gave reasonably valid estimates of the predictive performance with a small sample size [31]. The adjusted C-index values of the nomograms used to predict hospital survival and transplant-free survival were 0.72 (95% CI, 0.66–0.79) and 0.74 (95% CI, 0.68–0.79), respectively, after 1000 bootstrap cross-validation iterations. Collectively, the results strongly suggest that the two nomograms could provide prognostic information concerning survival for patients with HRS.

Our nomograms have value in clinical application. Firstly, our nomograms enrolled patients with HRS according to the latest diagnostic criteria of the International Club of Ascites in 2015 and took HRS as the target disease. The MELD/MELD–Na scores were applied for prognostic prediction in all end-stage liver diseases but not as specific targets for HRS. Secondly, we developed prognostic models to predict hospital survival and transplant-free survival, which were able to evaluate the prognosis of HRS more comprehensively. Both admitted patients with HRS and discharged patients with HRS saw benefits. Meanwhile, our models were convenient to use. The nomogram of hospital survival was based on the Child–Pugh score, and transplant-free survival was based on the MELD–Na score. Child–Pugh and MELD–Na scores are widely used to evaluate the condition of patients with liver cirrhosis, and they are easily accessible to clinicians.

The nomograms are commonly used to predict diagnosis, staging, and prognosis in cancer and different diseases. Nomograms are able to reduce statistical predictive models into a single numerical estimate of a clinical event probability for an individual patient, which facilitates patient–clinician communication and clinical decision making [32,33]. The main process of a nomogram includes construction, validation, and clinical utility. Most nomograms in medical journals are graphical or an equation; however, that is inconvenient to use and requires other auxiliary tools to calculate. The best future direction is one that combines a Web application with classical nomograms. Using shinyapps.io (https://gugle.shinyapps.io/, accessed on 4 May 2022), nomograms are written into a shiny application, and can be used by an individual patient on a smartphone

This study had certain limitations. Firstly, this was a single-center, retrospective cohort with a level of evidence lower than that of randomized controlled trials. Baseline population characteristics are indeed limitations of our study. Males accounted for a large proportion of the study population compared with females. Viral hepatitis-related cirrhosis is the most common cause of cirrhosis in China, while alcoholic cirrhosis is the most common cause of cirrhosis in Western countries. Thus, a large proportion of the patients included in our study were determined to have viral hepatitis-related cirrhosis. Expanding the sample size and conducting a multicenter study in the future could be an effective means to balance the bias and increase the credibility of our study. Secondly, although LT is the definitive therapy for HRS-AKI, kidney recovery after LT is not always universal. The mechanisms of it are not clear. We need to construct and study an LT cohort of patients with HRS to explore the independent risk factors associated with kidney recovery. Finally, although our two nomograms were internally validated using the bootstrap validation method, a future external validation in much larger cohorts from different centers or regions is needed to promote the efficacy and stability of nomograms.

## 5. Conclusions

In summary, in this study, we established a hospital survival nomogram and a transplant-free survival nomogram to predict the short-term and long-term mortality risks in patients with HRS. The two models had an excellent prediction accuracy and discriminatory ability. They might be useful as tools to help identify patients at high risk of HRS and to promote early intervention treatment.

## Figures and Tables

**Table 1:** Summary of demographics and clinical characteristics of patients with HRS.

Characteristics	Total (*n* = 149)
Age (year), mean ± SD	55.01 ± 12.71
Sex (male/female)	109/40
Causes of cirrhosis	
Viral hepatitis	65 (43.62%)
Alcoholic	48 (32.22%)
Others	36 (24.16%)
Mean arterial pressure (mmHg), median (MIN, MAX)	83 (52–121)
Albumin (g/L), mean ± SD	25.94 ± 4.75
Serum total bilirubin (μmol/L), median (MIN, MAX)	140.0 (9.5–815.0)
Serum sodium (mmol/L), median (MIN, MAX)	133.0 (110.7–150.1)
Prothrombin activity (%), mean ± SD	38.95 ± 18.68
Serum NH_3_ (μmol/L), median (MIN, MAX)	79 (16–483)
Spontaneous bacterial peritonitis	17 (11.40%)
Hemoglobin (g/L), median (MIN, MAX)	83 (50–145)
Clinical pattern of HRS (HRS-AKI/CKD)	128/21
Gastrointestinal bleeding	43 (28.86%)
Child–Pugh score (B class/C class)	20/129
MELD–Na score, median (MIN, MAX)	28 (11–51)
Baseline serum creatinine (μmol/L), median (MIN, MAX)	90.5 (34.5–354.2)
Peak serum creatinine (μmol/L), median (MIN, MAX)	211.5 (136.2–680.6)
Treatment strategy	
Terlipressin in combination with albumin	55 (36.91%)
Norepinephrine in combination with albumin	27 (18.12%)
Midodrine plus octreotide in combination with albumin	28 (18.79%)
Renal replacement therapy	26 (17.45%)
Liver transplantation	13 (8.72%)

Abbreviations: minimum number: MIN; maximum number: MAX.

**Table 2:** Cox proportional hazards regression analysis showing the association of variables with hospital survival and with transplant-free survival.

Variables	Hospital Survival				Transplant-Free Survival			
	Univariate		Multivariate		Univariate		Multivariate	
	HR (95%CI)	*p* Value	HR (95%CI)	*p* Value	HR (95%CI)	*p* Value	HR (95%CI)	*p* Value
Age	0.99 (0.98–1.01)	0.722			1.00 (0.98–1.02)	0.931		
Sex (female/male)	1.08 (0.66–1.77)	0.782			1.29 (0.82–2.03)	0.027	1.54 (0.95–2.50)	0.077
MAP (mmHg)								
>70 vs. ≤70	0.81 (0.42–1.54)	0.523			0.72 (0.39–1.32)	0.288		
Albumin (g/L)								
>30 vs. ≤30	1.76 (1.00–3.08)	0.048			1.68 (0.99–2.87)	0.057		
Total bilirubin (µmol/L)								
>110 vs. ≤110	2.05 (1.26–3.36)	0.004			2.24 (1.44–3.48)	<0.001		
Serum sodium (mmol/L)								
>130 vs. ≤130	1.07 (0.66–1.74)	0.79			0.89 (0.572–1.38)	0.604		
Prothrombin activity (%)								
>27 vs. ≤27	0.34 (0.22–0.55)	<0.001	0.44 (0.27–0.73)	0.001	0.29 (0.18–0.45)	<0.001	0.41 (0.22–0.77)	0.006
Serum NH_3_ (µmol/L)								
>100 vs. ≤100	1.56 (0.96–2.51)	0.071			1.71 (1.09–2.68)	0.020		
Spontaneous bacterial peritonitis								
Yes vs. no	0.77 (0.37–1.62)	0.492			0.77 (0.40–1.50)	0.451		
Hemoglobin (g/L)								
>60 vs. ≤60	1.11 (0.62–1.99)	0.721			0.94 (0.56–1.58)	0.821		
HRS clinical pattern								
HRS-CKD vs. HRS-AKI	0.52 (0.24–1.13)	0.009	0.45 (0.202–1.023)	0.056	0.55 (0.36–1.22)	0.002	0.51 (0.26–0.98)	0.045
Gastrointestinal bleeding								
Yes vs. no	1.27 (0.79–2.02)	0.310			1.52 (0.98–2.34)	0.039		
Child–Pugh class								
C vs. B	1.17 (0.58–2.35)	0.0001	1.49 (0.83–2.68)	0.045	1.31 (0.697–2.47)	0.0801		
MELD–Na score								
21–30 vs. ≤20	1.45 (0.69–3.05)	0.033			1.67 (0.87–3.19)	0.001	1.78 (0.92–3.44)	0.078
>30 vs. ≤20	3.56 (1.70–7.45)	0.024			4.46 (2.30–8.63)	<0.001	2.27 (1.02–5.07)	0.005
Baseline serum creatinine (µmol/L), log10	1.70 (0.584–4.96)	0.0034	2.18(0.72–6.62)	0.050	1.31 (0.49–3.53)	0.019		
Peak serum creatinine (µmol/L), log10	NA	NA	NA	NA	8.32 (3.02–22.97)	<0.001	14.72 (4.66–46.51)	<0.001
Vasoconstrictor treatment								
Norepinephrine with albumin vs. terlipressin with albumin	1.35 (0.76–2.66)	0.407			1.00 (0.54–1.85)	0.997		
Midodrine plus octreotide with albumin vs. terlipressin with albumin	2.11 (1.09–4.09)	0.527			1.61 (0.88–2.93)	0.124		

Abbreviations: MAP: mean arterial pressure; HRS: hepatorenal syndrome; CKD: chronic kidney disease; AKI: acute kidney injury; CI: confidence interval; HR: hazard ratio; MELD–Na: model for end-stage liver disease–sodium.

**Table 3:** Comparison between actual survival probability and predicted survival probability.

		Actual Survival Probability Based on Kaplan–Meier Curves	Predicted Survival Probability Calculated from Nomograms
Hospital survival			
15d	High-risk group	40.70% (95% CI, 28.03–59.20%)	41.25% (95% CI, 33.57–48.89%)
	Low-risk group	83.40% (95% CI, 76.06–91.50%)	82.71% (95% CI, 80.93–85.33%)
30d	High-risk group	14.00% (95% CI, 6.32–31.00%)	14.63% (95% CI, 9.41–19.84%)
	Low-risk group	64.60% (95% CI, 54.23–76.90%)	63.70% (95% CI, 59.06–68.34%)
45d	High-risk group	7.00% (95% CI, 1.98–24.7%)	5.74% (95% CI, 2.74–8.74%)
	Low-risk group	46.30% (95% CI, 33.37–64.20%)	49.36% (95% CI, 44.24–52.23%)
Transplant-free survival			
6m	High-risk group	NA †	11.33% (95% CI, 6.61–16.06%)
	Low-risk group	53.4% (95% CI, 40.33–70.70%)	56.50% (95% CI, 48.67–64.32%)
12m	High-risk group	NA	2.04% (95% CI, 0.09–3.98%)
	Low-risk group	30.80% (95% CI, 16.55–57.40%)	31.69% (95% CI, 22.93–40.52%)
18m	High-risk group	NA	0.73% (95% CI, 0.00–1.88%)
	Low-risk group	23.10% (95% CI, 9.97–53.60%)	27.59% (95% CI, 19.03–36.16%)

Note: †: The follow-up range of the patients in the high-risk group who died was 0.03–2.48 months.

## Data Availability

The data that support the findings of this study are available on request from the corresponding author.

## Supplementary Materials

The following supporting information can be downloaded at: https://www.mdpi.com/article/10.3390/diagnostics12061417/s1, Figure S1: Schoenfeld test for predicting hospital survival nomogram and transplant-free survival.
