# Supplementary figures and images for: Prognostic Nomograms for Hospital Survival and Transplant-Free Survival of Patients with Hepatorenal Syndrome: A Retrospective Cohort Study
Source: Diagnostics (Basel). 2022 Jun 8;12(6):1417. doi: 10.3390/diagnostics12061417 (PMC9221587; doi:10.3390/diagnostics12061417)

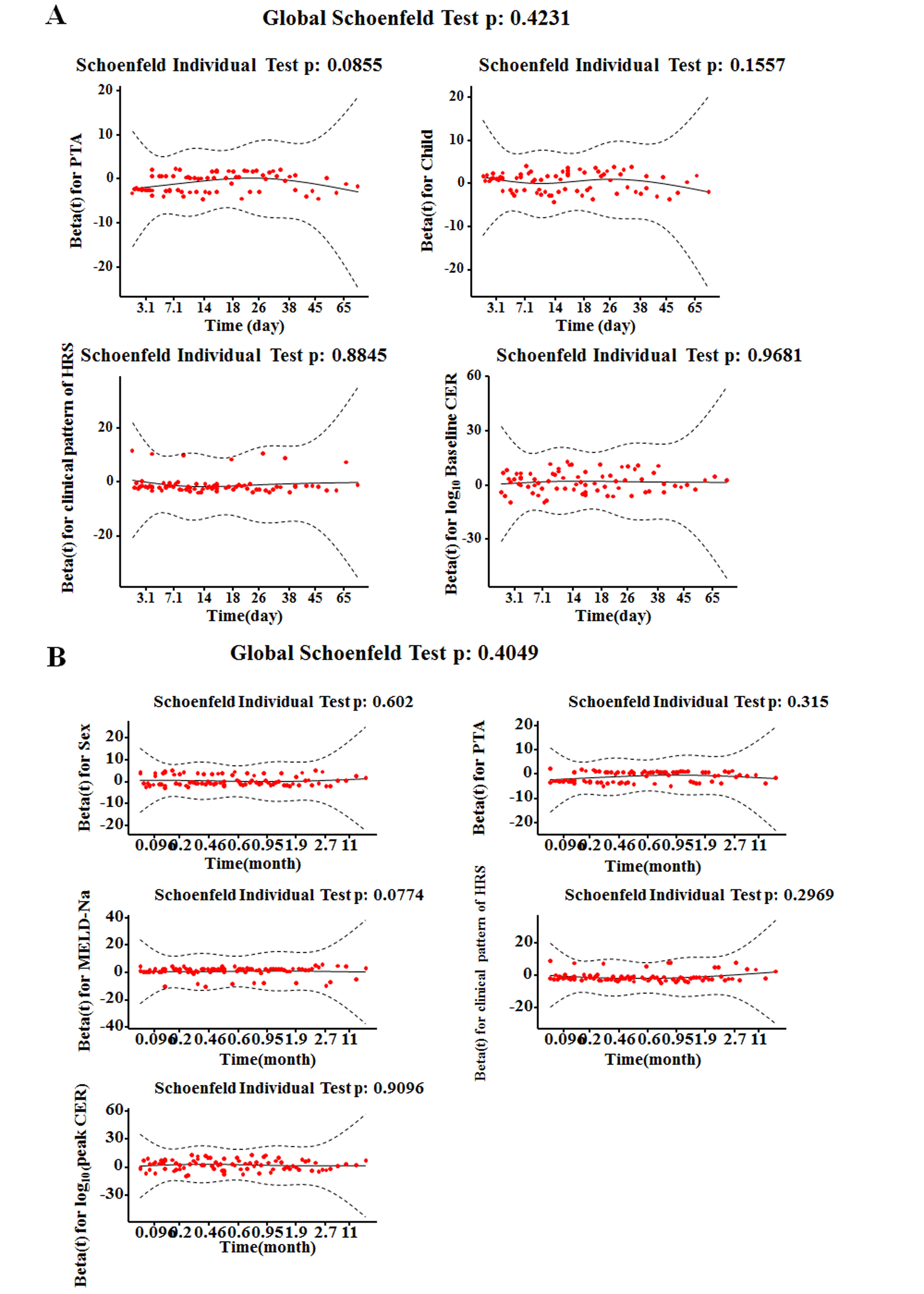

Supplement: Supplementary file 1 [file diagnostics-12-01417-s001.zip › Figure S1.tif]
